# Thermal Reduction of Graphene Oxide Mitigates Its In Vivo Genotoxicity Toward *Xenopus laevis* Tadpoles

**DOI:** 10.3390/nano9040584

**Published:** 2019-04-09

**Authors:** Lauris Evariste, Laura Lagier, Patrice Gonzalez, Antoine Mottier, Florence Mouchet, Stéphanie Cadarsi, Pierre Lonchambon, Guillemine Daffe, George Chimowa, Cyril Sarrieu, Elise Ompraret, Anne-Marie Galibert, Camélia Matei Ghimbeu, Eric Pinelli, Emmanuel Flahaut, Laury Gauthier

**Affiliations:** 1 EcoLab, Université de Toulouse, CNRS, INPT, UPS, 31400 Toulouse, France; laura.lagier@hotmail.com (L.L.); antoine.mottier@ensat.fr (A.M.); florence.mouchet@ensat.fr (F.M.); stephaniecad@gmail.com (S.C.); pinelli@ensat.fr (E.P.); laury.gauthier@univ-tlse3.fr (L.G.); 2 Univ. Bordeaux, UMR EPOC CNRS 5805, Aquatic ecotoxicology team, 33120 Arcachon, France; patrice.gonzalez@u-bordeaux.fr (P.G.); emmanuel.flahaut@univ-tlse3.fr (E.F.); 3 CIRIMAT, Université de Toulouse, CNRS, INPT, UPS, UMR CNRS-UPS-INP N°5085, Université Toulouse 3 Paul Sabatier, Bât. CIRIMAT, 118 route de Narbonne, 31062 Toulouse CEDEX 9, France; lonchambon@chimie.ups-tlse.fr (P.L.); gchimowa11@gmail.com (G.C.); cyril.sarrieu@gmail.com (C.S.); eompraret@gmail.com (E.O.); galibert@chimie.ups-tlse.fr (A.-M.G.); 4 CNRS, Universite de Bordeaux, Observatoire Aquitain des Sciences de l’Univers, UMS 2567 POREA, Allee Geoffroy Saint Hilaire, F-33615 Pessac, France; guillemine.daffe@u-bordeaux.fr; 5 Institut de Science des Matériaux de Mulhouse (IS2M), UMR 7360 CNRS—UHA, 15 rue Jean Starcky, BP 2488, 68057 Mulhouse CEDEX, France; camelia.ghimbeu@uha.fr

**Keywords:** graphene oxide, reduced graphene oxide, micronucleus, oxidative stress, safer-by-design

## Abstract

The worldwide increase of graphene family materials raises the question of the potential consequences resulting from their release in the environment and future consequences on ecosystem health, especially in the aquatic environment in which they are likely to accumulate. Thus, there is a need to evaluate the biological and ecological risk but also to find innovative solutions leading to the production of safer materials. This work focuses on the evaluation of functional group-safety relationships regarding to graphene oxide (GO) in vivo genotoxic potential toward *X. laevis* tadpoles. For this purpose, thermal treatments in H_2_ atmosphere were applied to produce reduced graphene oxide (rGOs) with different surface group compositions. Analysis performed indicated that GO induced disturbances in erythrocyte cell cycle leading to accumulation of cells in G0/G1 phase. Significant genotoxicity due to oxidative stress was observed in larvae exposed to low GO concentration (0.1 mg·L^−1^). Reduction of GO at 200 °C and 1000 °C produced a material that was no longer genotoxic at low concentrations. X-ray photoelectron spectroscopy (XPS) analysis indicated that epoxide groups may constitute a good candidate to explain the genotoxic potential of the most oxidized form of the material. Thermal reduction of GO may constitute an appropriate “safer-by-design” strategy for the development of a safer material for environment.

## 1. Introduction

Carbon-based nanomaterials (CBMs) and especially 2D materials related to graphene [1] possess unique properties [2,3], triggering high expectations for the development of new technological applications and are forecasted to be produced at industrial-scale [4]. Among these graphene-based materials (GBMs), graphene derivatives such as graphene oxide (GO) and reduced graphene oxide (rGO) appear as very attractive due to their high stability after dispersion in various solvents, facilitating handling and processing of graphene-containing nanocomposites [5,6]. To ensure the safe and sustainable development of this innovative technology, evaluation of its biological and ecological risk, as well as finding innovative solutions to mitigate the hazard potential, are essential [7,8,9]. The increasing GBMs production raises concerns over their release into the environment, where it is likely to occur at any stage of the material life cycle [10,11,12]. However, compared to the increasing number of studies dealing with GBMs synthesis processes or application development advances, relatively few are devoted to studying their toxicity, and even less to their ecotoxicity. Due to its hydrophilic properties associated with the presence of oxygen-containing functional groups at their surface, GO and rGO could potentially be highly reactive towards multiple components of the environment [13]. Moreover, different physico-chemical behaviours between GO and rGO can be expected in the environment [14], associated with changes occurring in surface functional groups during the reduction process [15].

Most of the GBMs toxicological data available were obtained through in vitro experiments focusing on cytotoxicity towards mammalian cells [16,17,18]. For instance, some studies demonstrated that oxidized graphene-based nanoparticles exerted higher toxicity compared to their reduced counterparts [19,20,21], while others obtained contradictory results, indicating higher toxicity exerted by the reduced form of GO [22,23]. Among possible toxic effects, genotoxicity may have non-negligible consequences because unrepaired and/or improperly repaired DNA damage may in turn cause cellular dysfunctions and tumor formation, leading to the death of organisms [24] and further decline of a population [25]. However, it was pointed out that in vivo genotoxic potential of GBMs is still relatively poorly investigated [16,26,27]. In vivo experiments focusing on genotoxicity were mainly performed in rodents microinjected with nanomaterials [28,29,30] and data are remaining scarce particularly for aquatic species. A study performed using the comet assay in zebrafish failed to highlight genotoxic effects into fish gills after short-term exposure to GO [31]. Since the data available are contradictory, there is a need to clarify in vivo genotoxic potential and toxicological mechanisms associated to GO and rGO exposure, in order to fill persisting knowledge gaps concerning their eco-genotoxicity [32]. Amphibians are widely used for ecotoxicological studies and are recognized as sensitive organisms to genotoxic compounds, especially at the larval stage [33,34]. Tadpoles of the African clawed frog, *Xenopus laevis*, have previously been used for assessment of raw carbon-based nanoparticles ecotoxicity [35,36,37,38] and adverse effects on larval growth were previously reported after GO exposure [39].

The aim of the present work is to assess the in vivo genotoxic potential of GO in *X. laevis* as well as understanding toxicological pathways involved in the genotoxic response after exposure to a commercial form of the material. To determine the implication of the oxidation degree and surface functions in the toxicological response, thermal treatments in H_2_ atmosphere were applied at two temperatures (200 °C and 1000 °C) to produce rGO exhibiting different surface functions. The complete characterization of the tested materials was performed to identify the role of functional groups in the genotoxic response.

## 2. Materials and Methods

### 2.1. Synthesis and Characterization of Graphene Oxide and Reduced Graphene Oxide

**Figure 1:**
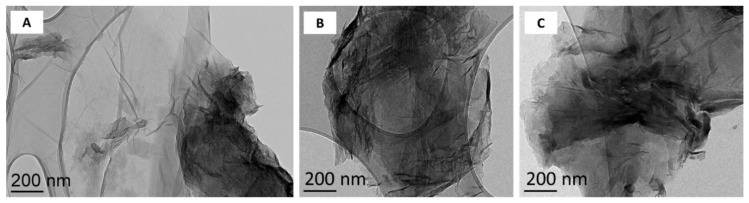
Transmission electron microscopy micrographs of (**A**) Graphene oxide, (**B**) reduced graphene oxide at 200 °C, (**C**) reduced graphene oxide at 1000 °C.

Graphene oxide was provided by Antolin Group and prepared by oxidation of Grupo Antolin Carbon Nanofibers (GANF^®^)(Grupo Antolín, Burgos, Spain) using the Hummer’s method [40,41]. Tested rGO resulted from reduction of this GO in H_2_ atmosphere with a hydrogen flow rate of 5 L·h^−1^ at 200 °C (rGO200) or 1000 °C (rGO1000). Reduction was performed under controlled conditions to modify the oxidation level with minimal impact on material morphology, lateral size, and number of layers (Figure 1). Reduction produced rGO samples with closely related physico-chemical characteristics compared to the starting GO material, except for their surface chemistry and their wetting properties. Physico-chemical characteristics of the tested materials are detailed in Table 1. Reduction at 200 °C was only partial and allowed keeping most of the oxygen in the material, while the reduction at 1000 °C almost completely removed the oxygen.

Elemental analysis (percentage of O and C atoms) was obtained by X-ray photoelectron spectroscopy (XPS). X-ray photoelectron spectroscopy (XPS) spectra were recorded with a VG SCIENTA SES-2002 spectrometer (Scienta Omicron, Taunusstein, Germany) equipped with a concentric hemispherical analyzer. Specific surface area was determined by N_2_ adsorption according to the Brunauer, Emett and Teller’s theory (BET) on dry powdered samples using a Micrometrics Flow Sorb II 2300 (Micromeritics, Norcross, GA, USA). The dispersion behavior of the two nanomaterials was analyzed in the exposure medium using a Turbiscan™ LAB Stability Analyzer (Formulaction SA, Toulouse, France). Transmission and backscattering of the near infrared light source (880 nm) was measured every 40 µm of the sample height. In order to ensure the detection of the nanoparticles, the concentration of 10 mg·L^−1^ of GO and rGO was selected for dispersion monitoring.

### 2.2. Metals and Polycyclic Aromatic Hydrocarbons (PAHs) Concentration Analysis in Graphene Oxide

GO from Grupo Antolin was prepared by oxidation of GANF^®^ (grupo Antolin carbon nanofibers) which synthesis involve Ni, Co, Fe and Mn as metal catalysts. In addition, PAHs could be associated with GO because of their possible generation during GANF and GO synthesis and may be released by desorption from carbon nanomaterials in water [44]. The possible presence of these compounds was checked to avoid misanalysis of toxicity-related results [44]. Quantification of metal residues in mineralized GO powder was performed as described by Ayouni-Derouiche et al. [45] using ICP AES, iCAPTM 6300 analyzer (Thermo Fisher Scientific, Germany) (Crealins, Lyon, France). 32 PAHs compounds were analysed from GO dispersion in deionized water using gas chromatography-mass spectrometry (GC-MS) according to the normalized procedure NF ISO 28,540 (MicroPolluants Technologie S.A., Saint-Julien-lès-Metz, France).

### 2.3. Xenopus Rearing, Breeding and Exposure Conditions

*Xenopus* rearing and breeding were described in previous works [35,36]. Briefly, spawning of sexually mature *Xenopus* was induced by injection of pregnant mare’s gonadotropin. Fecundated eggs obtained were bred in active charcoal filtered tap water at 22 ± 2 °C and fed with ground aquarium fish food (TetraPhyll^®^, Tetra, Melle, Germany) until they reach stage 50 according to Nieuwkoop & Faber development table [46]. Groups of 20 larvae were exposed for 12 days under semi-static conditions with daily feeding and exposure media renewal following the international standard ISO 21427-1 procedure. Negative control (NC) condition was composed of reconstituted water (RW; 294 mg·L^−1^ CaCl_2_·2H_2_O; 123.25 mg·L^−1^ MgSO_4_·7H_2_O; 64.75 mg·L^−1^ NaHCO_3_; 5.75 mg·L^−1^ KCl) and cyclophosphamide monohydrate ([6055-19-2], Sigma-Aldrich Chimie, Saint-Quentin Fallavier, France) at 20 mg·L^−1^ in RW was used as genotoxic positive control (PC). GO tested concentration ranged from 0.1 to 50 mg·L^−1^. Due to significant genotoxic effects induced by GO at the concentration of 0.1 mg·L^−1^, this concentration was chosen to further determine toxicological pathways involved as well as to determine the consequences of thermal reduction on toxicity. Thus, rGO200 and rGO1000 were only tested at 0.1 mg·L^−1^.

### 2.4. Micronucleus Test and Cell Cycle Analysis

After 12 days of exposure, blood samples were obtained by cardiac puncture in *Xenopus* larvae anaesthetized by immersion in MS222 solution at 0.1 g·L^−1^. For micronuclei assay, smears were prepared from blood samples, fixed in methanol for 10 min before performing hematoxylin and eosin staining. The number of micronucleated erythrocytes (MNE) over a total of 1000 erythrocytes (MNE ‰) was counted under the optical microscope Olympus CX41 (oil immersion lens, ×1500) (Olympus, Tokyo, Japan). Blood sub-samples were fixed using cold ethanol (70% v/v) and stored at −20 °C until use. Prior to the flow cytometric analysis, cells were rinsed using PBS and labelled with FxCycle™ PI/RNase Staining Solution (Thermo Fisher Scientific, Bremen, Germany) according to manufacturer’s recommendations. Propidium iodide fluorescence was measured using MACSQuant analyzer 10 (Miltenyi Biotec, San Diego, CA, USA) equipped with a 488-nm excitation laser. For each sample, 10,000 events were acquired in a region corresponding to erythrocytes after removal of cell doublets. For gating strategy, see Figure S1.

### 2.5. Gene Expression Analysis in the Livers

As the liver constitute the main organ of erythropoiesis and is implied in multiple metabolic functions in *X. laevis* [47], this organ was chosen to determine toxicological mechanisms involved in the genotoxic response at low GBMs concentration (0.1 mg·L^−1^). For this purpose, analysis of the expression of 15 genes encoding for proteins involved in oxidative stress response (gpx1, cat, sod (Cu/Zn), sod(Mn)), inflammation processes (pparγ, cox1, cox2, lta4, 5-lox), detoxification (cyp1a1, tap, gst) and DNA repair (rad51, mutl, odc) was performed. Total RNA were extracted from 15 to 25 mg of liver samples using the SV Total Isolation System kit (Promega, Madison, WI, USA) according to manufacturer’s instructions. Reverse transcription was carried out from 1 µg of total RNA using the GoScriptTM Reverse Transcription System kit (Promega) according to manufacturer’s recommendations. Nnucleotide sequences of the primers were obtained from the online NCBI Nucleotide database and primer pairs were determined using the Primer3Plus software. All the primer pairs used are reported in Table S1.

Real-time qPCR was carried out using GoTaq^®^ qPCR Master Mix kit (Promega, Madison, WI, USA) on five samples per condition. PCR reactions contained 17 µL of a mixture of Nuclease-Free Water and GoTaq^®^ qPCR Master Mix containing the SyberGreen fluorescent dye, 2 µL of specific primer pairs mix (200 µM each) and 1µL of cDNA. Real-time quantitative PCR reactions were performed in a Mx3000P^®^ qPCR System (Stratagene, La Jolla, CA, USA). The amplification program consisted in one cycle at 95 °C for 10 min, then 45 amplification cycles at 95 °C for 30 s, 60 °C for 30 s and 72 °C for 30 s. Specificity was determined for each reaction from the dissociation curve of the PCR product. This dissociation curve was obtained by following the SYBR Green fluorescence level during a gradual heating of the PCR products from 60 to 95 °C.

Cycle thresholds (Ct) were obtained from MxProTM qPCR software for each gene. Relative quantification of each gene expression level was normalized according to the mean Ct value of two stable reference genes (β actin, gapdh) according to the 2∆Ct methods described by Livak and Schmittgen [48]. Induction factors, compared to control group, were then determined as previously described [49].

### 2.6. Statistical Analysis

Data of micronucleus frequencies from three repeated experiments were analyzed using McGill non-parametric test [50] on median values of each group of larvae. This test consists in comparing medians of samples of size n (where n ≥ 7) and in determining their 95% confidence intervals (95% CI). 95% CI are expressed by M ± 1.57 × IQR/√n, where M is the median and IQR is the inter-quartile range [50]. The difference between the medians of the test groups and the median of the NC group is significant with 95% certainty if there is no overlap. For cell-cycle data, normality was assessed with Kolmogorov-Smirnov test and homogeneity of variances with Levene’s test. One-way analysis of variance (ANOVA) followed by Tukey test were used to compare cell-cycle phase distribution among conditions. One-way ANOVA on ranks and Tukey post-hoc test (*p* < 0.05) were used to statistically compare differential gene expression levels.

## 3. Results and Discussion

### 3.1. Surface Chemistry and Dispersion Behavior

**Figure 2:**
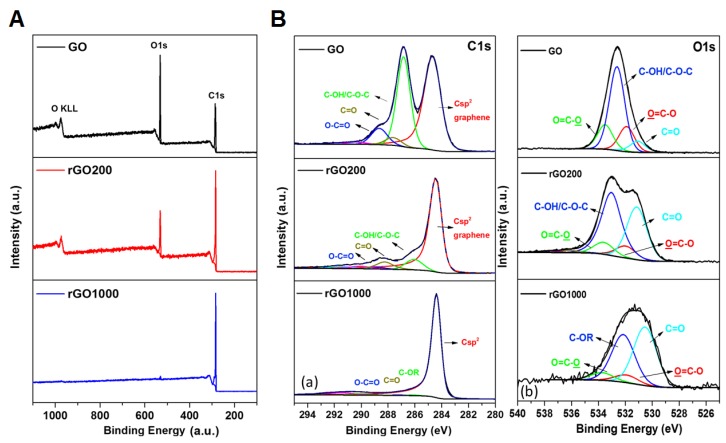
X-ray photoelectron spectroscopy (XPS) survey spectra of GO, rGO200 and rGO1000 materials (**A**); C1s and O1s deconvoluted XPS spectra for GO, rGO200 and rGO1000 (**B**).

Surface chemistry of GO and rGOs evaluated by high resolution X-ray photoelectron spectroscopy (XPS) allowed identification of oxygen-containing groups present in the materials (Figure 2, Table 2).

For GO, the C1s spectrum obtained by XPS exhibits two main peaks at 284.6 eV and 286.8 eV (Figure 2B), which are correlated with the sp2 carbon (Csp2) of the graphene and oxygen functional groups, respectively. The 286.8 eV signal is deconvoluted into several peaks located at 286.8 eV, 288.6 eV and 287.61 eV. The most intense one is the 286.8 eV peak (24.7 at. %, Table 2), which corresponds to the carbon involved in hydroxyl groups (C-OH), ether and particularly epoxide groups (C–O–C) [51,52].

The two other peaks are related to carbonyl (C=O) and carboxylic (O=C–O) groups and account for 2.5 and 5.3 at. %, respectively. Thermal reduction induced modifications of the chemical composition of GO. After annealing GO at 200 °C, the C1s spectra exhibited mainly one peak at 284.6 eV, corresponding to the sp2 carbon in graphene. This peak was narrower compared to that of GO, suggesting an increase in the graphitization level. The intense peak of the epoxide groups (286.8 nm) present on GO was removed by this treatment, leaving two shoulders associated with the hydroxyl groups and, to a lesser extent, with the ethers/epoxide (C–OH/C–O–C), C=O and O=C–O oxygen-containing functional groups. In agreement with previous work from Jung et al. [51], the use of temperature programmed desorption coupled with mass spectrometry (TPD-MS) indicated that removal of epoxide groups at 200 °C was accompanied by the release of CO, CO2 and H2O gases (data not shown). A dual path mechanism which proceeded by the release of solely molecular oxygen via a cycloaddition reaction from epoxide–epoxide pairs was proposed for the reduction of oxidized graphene [52]. Formation of ether–epoxide pairs at high O coverage further promoted the elimination of oxygen functional groups by releasing CO/CO2 mixtures, along with H_2_O formation. Stronger reduction conditions (1000 °C) resulted in a material containing poorly oxygenated surface groups (Table 2), and a total oxygen content of 1.5 at. %. Details about the nature of the oxygen groups can also be seen in the O1s spectrum (Figure 2B).

**Figure 3:**
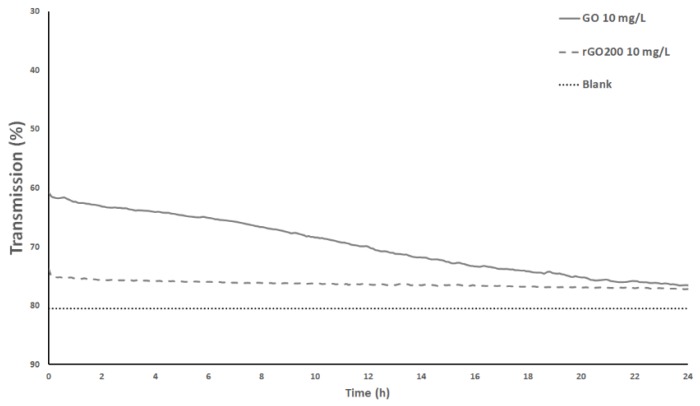
Monitoring of the stability of GO and rGO200 dispersion in the water column of exposure medium over 24 h (in absence of *Xenopus* larvae), expressed by the percentage of transmission detected after the light goes through the sample. Blank: medium without nanoparticles.

The dispersion over time of GO and rGO200 in the medium of exposure (reconstituted water composed of deionized water added with salts) and in absence of Xenopus larvae is shown in Figure 3.

The results indicate a slight decrease in transmission over 24 h for the GO while this was not observed for rGO. Dispersion of these two materials was previously studied and a good dispersion capacity of both materials was observed in distilled water, with a better stability in the case of GO [53,54]. However, it was indicated that the presence of CaCl_2_ reduced the GO stability due to adsorption of Ca^2+^ ions on the negatively charged functional groups, leading to reduced surface charge. In addition, rGO stability was less influenced by these ions due to the lower amount of functional groups, limiting Ca^2+^ adsorption [55]. As our exposure medium contains NaCl and CaCl_2_, results from Chowdhury and collaborators [55] are consistent with our observations. Nonetheless, the dispersion state of the nanoparticles is strongly affected in presence of Xenopus larvae. As active filter feeders, it was previously observed that the water column is completely filtered in less than 24 h, resulting in nanoparticle accumulation in feces [39].

### 3.2. Metals and PAHs Contamination

Results of metallic residue quantifications are expressed in milligrams of metal per liter of exposure medium (Table 3).

According to these results, at 10 mg·L^−1^ of GO dispersed in medium, the concentration of metals was 6.9 × 10^−4^, <2.3 × 10^−4^, <6.0 × 10^−4^ and 149.8 × 10^−4^ mg·L^−1^ for Ni, Co, Fe and Mn, respectively. However, a recovery efficiency of 91 ± 5% was measured from the certified reference material leading to a slight under-estimation of metal quantities in GO due to the complexity to perform metal analysis in a nanocarbon matrix. Among 32 PAHs compounds investigated in the exposure medium contaminated with GO at 10 mg·L^−1^, 24 were below detection limit (<20 ng·L^−1^). PAHs concentrations ranged from 2.4 × 10^−4^ µg·L^−1^ for Fluoranthene and Benzo(a)anthracene to 5.8 × 10^−4^ µg·L^−1^ for 2-Methyl Naphtalene (Table 3). After 12 days of exposure to such GO concentration, the total amount of contaminants potentially bioavailable for Xenopus larvae would be 16.6 × 10^−3^, <5.52 × 10^−3^, <14.4 × 10^−3^ and 359.52 × 10^−3^ µg of Ni, Co, Fe and Mn, respectively and a total amount of 5.95 × 10^−2^ µg of PAHs. Metal ions such as Mn^2+^ and Fe^2+^ were shown to induce DNA scission when associated to GO [56] and PAHs constitute hazardous contaminants for humans and wildlife [57,58] that are known to exert genotoxicity towards amphibians [59,60]. However, total concentrations detected were too low to induce significant toxicity in larvae [61]. Thus, we can state that results obtained from bioassays performed in our study could be fully attributed to GO exposure.

### 3.3. Cell-cycle Analysis

**Figure 4:**
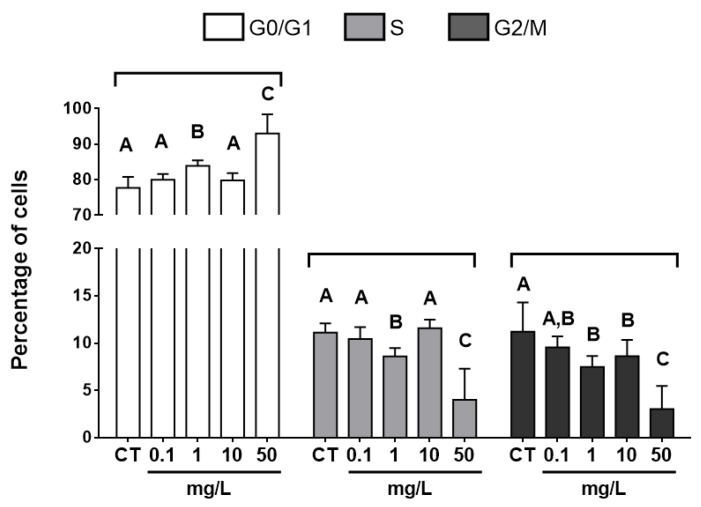
Cell-cycle distribution in G0/G1, S and G2/M phase analyzed from circulating erythrocytes of *Xenopus laevis* exposed to increasing concentrations of GO for 12 days. NC: negative control, N = 13, analysis of variance (ANOVA) *p* < 0.001 followed by Tukey test. Letters indicate significant differences between concentrations tested for each phase of the cell cycle.

Flow cytometry measurement of erythrocyte cell cycle highlighted an overall significant decrease in G2/M and S-phase cells (ANOVA, S-phase: *p* < 0.001; G2/M: *p* < 0.001) as well as an increase in G0/G1 cells (ANOVA, *p* < 0.001) with increasing concentration of GO (Figure 4).

The lowest concentration inducing significant changes in erythrocyte cell cycle was observed at 1 mg·L^−1^ of GO, resulting in significantly decreased G2/M and S-phase compared to the control group, while results obtained from organisms exposed at 0.1 mg·L^−1^ of GO were similar to the control group. After 12 days of exposure at the concentration of 50 mg·L^−1^, erythrocyte accumulated in the G0/G1 phase of cell-cycle with a concomitant strong decrease in G2/M and S-phase percentage. According to data from the literature, almost all studies focusing on effects of GO on cell-cycle-related endpoints were performed in vitro. However, exposures to pristine or functionalized graphene oxide were shown to disturb cell-cycle progression, leading to accumulations of cells in early phases [62,63,64,65] that is consistent with our results. Petibone and collaborators [64] highlighted the key role of the p53 protein in cell cycle arrest after GO exposure, leading to cell accumulation in G0/G1 phase while p53-deficient cell line accumulated in S-phase. p53 is known to be involved in DNA damage response signaling pathway, driving to cell cycle arrest following genotoxic stress [66,67]. Upregulation of p53 expression was previously highlighted in mouse embryonic stem cells after exposure to other carbon-based nanoparticles such as carbon nanotubes and nanodiamonds [68,69]. In addition, a downregulation of protein S-phase kinase-associated protein involved in the control of the progression from G1 phase to S-phase during mitosis process was observed in human liver cancer HepG2 cells exposed to graphene oxide [70]. As the liver constitutes the main organ of erythropoiesis in *X. laevis* [47], a closely related mechanism could explain results obtained in our study. Thereby we can suggest that disturbances of erythrocyte cell-cycle observed in vivo in *X. laevis* tadpoles under our experimental conditions could be associated to modulations of gene expression and activities of proteins involved in cell-cycle regulation. However, further studies are needed to confirm these hypotheses.

Cell division constitutes a sine qua non-condition to produce a micronucleus after chromosome breakage (clastogenesis) or/and disturbance of chromosome segregation machinery (aneugenesis) [71,72]. As the majority of cells were blocked at the G0/G1 stage in larvae exposed to GO at concentrations ranging from 1 to 50 mg·L^−1^, the decrease in erythrocyte mitotic rates measured would lead to inconsistent results in the evaluation of micronuclei induction at these concentrations.

### 3.4. Genotoxicity

**Figure 5:**
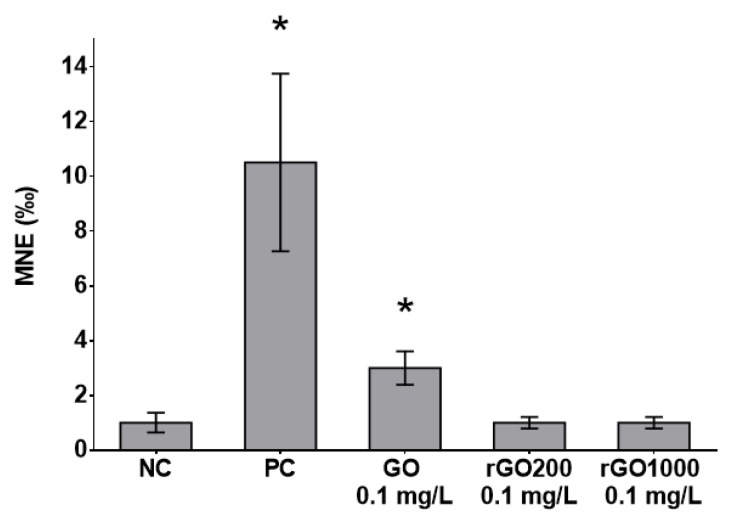
Micronucleus induction measured in erythrocytes of *Xenopus laevis* larvae exposed for 12 days to GO or rGO (rGO200 or rGO1000). MNE: micronucleated erythrocytes; NC: negative control; PC: positive control; *: significant difference compared to the NC (McGill test).

In accordance with ISO/FDIS 21427-1 standards, as mitotic rates were significantly lower in the 1, 10 and 50 mg·L^−1^ of GO compared to the control group, micronuclei were not accounted in these conditions. In addition, in all experimental groups, larvae exposed to PC (cyclophosphamide at 20 mg·L^−1^) exhibited significantly higher median values of micronucleated erythrocytes (MNE ‰) compared to their respective NC group, validating the results of the micronucleus tests. A significant increase in micronucleus occurrence was observed in erythrocytes of larvae exposed to the 0.1 mg·L^−1^ concentration of GO compared to the NC group (Figure 5). Contrary to the results obtained with the most oxidized form of the material, exposure to 0.1 mg·L^−1^ of rGO did not induce an increase in micronucleated erythrocyte occurrence compared to the control group, regardless of the reduction temperature performed (200 °C or 1000 °C, Figure 5).

The results obtained demonstrated that GO is able to induce the formation of nuclear abnormalities in vivo at low concentrations in amphibian larvae. Induction of genotoxic effects through increase of micronuclei occurrence as well as DNA fragmentation or chromosome aberration were previously observed in vivo in rodents [28,29,73,74,75]. Thus, these data from the literature are consistent with our results. On the contrary, no genotoxicity using comet assay was found in the gills of zebrafish exposed to GO concentrations from 2 to 20 mg·L^−1^ during 72 h [31]. However, despite the differences in exposure duration and conditions, micronucleus and comet assays are not devoted to highlighting similar genotoxic pathways and mechanisms [76]. This also suggests that GO exposure induces breaks at chromosomal level rather than chromatic level [71]. It was highlighted that GO strongly interacted with DNA in vitro, causing interferences with DNA segregation during cell-cycle and generated mutagenic effects [29,77]. In addition, according to molecular dynamics simulations, the driving force of interactions between nucleotides and carbon-based nanosurfaces is the π stacking noncovalent interaction between aromatic rings, which may lead to self-assembly between DNA and graphene potentially causing DNA deformation and breakage [78]. However, this assumption is unlikely due to more limited direct interactions between GO and erythroid progenitors or circulating erythrocytes using in vivo exposure. Thus, it is more likely that mutagenic effects observed were associated to DNA damages generated by reactive oxygen species (ROS) that are described as being mainly implied in DNA fragmentation, as they are involved as secondary messengers in many intracellular signaling cascades and can damage cellular macromolecules [79].

### 3.5. Genes Expressions in the Livers of Larvae Exposed to GO and rGO

Analysis of the relative gene expression levels after 12 days of exposure reveals a significant induction of most of the studied genes in the liver of larvae exposed to GO at 0.1 mg·L^−1^ (Table 4). Some genes involved in oxidative stress response and inflammation (gpx1, sod(Cu/Zn), sod(Mn), pparγ, 5-lox and cox1) were significantly induced from 2.6 to 5.84 times more than the negative control. Finally, some detoxification processes occurred as shown by the induction of cyp1a1 and tap. On the contrary, no significant modulation of gene expressions was noticed in rGO conditions (Table 4).

Upregulation of gene expression related to cytoplasmic and mitochondrial super-oxide dismutase (sod(Cu/Zn) and sod(Mn)) indicates that exposure to 0.1 mg·L^−1^ of GO induce oxidative stress. Furthermore, contrary to RNA expression level of the catalase gene, significant upregulation of gpx1 suggest that hydrogen peroxide produced is mainly eliminated through glutathione pathway [80,81]. Capacity of GO to induce oxidative stress in vivo was observed in a wide range of biological models such as rodents [28], fish [82], nematodes [83] or paramecium [84]. In the case of carbon nanotube exposure, an interdependent relationship between ROS production and inflammatory response was evidenced [85]. Similarly, inflammatory responses were frequently observed in vivo in rodents after GO exposure [86,87,88,89] and to a lesser extent after rGO exposure [21,90]. This corroborate with our results and confirm previous hypothesis suggesting that observed genotoxicity result from oxidative stress and inflammation process in the liver [91], constituting the erythropoietic organ in *X. laevis* tadpoles. Thus, oxidative stress affecting erythrocyte progenitors associated to an absence of upregulation of DNA repair-related genes result in the release of micronucleated erythrocytes in the circulation.

Thermal reduction under a hydrogen atmosphere produced material that no longer exerted oxidative stress, inflammatory response, disturbance of erythrocyte cell cycle (data not shown) as well as genotoxicity at low concentration. Genotoxic potential of GO in vitro was previously shown to be related to material lateral size [92]. However, in our study conditions, GO and rGOs tested were of similar range of lateral size suggesting that differences observed between the two types of GBMs were not correlated to this material characteristic. The main difference between tested GO and rGOs was the oxygen content (C/O ratio) and by extension surface chemistry including the nature of oxygen-containing functionalities. This parameter appears to be a good candidate to explain differences observed in genotoxic potential of these nanomaterials. Indeed, some studies demonstrated that oxidized carbon-based nanoparticles exerted higher genotoxicity compared to their non-oxidized counterparts [20,93]. However, other studies obtained contradictory results, indicated that the reduced form of GO exerted higher toxicity compared to the oxidized material. It was observed for cytotoxicity in cell lines [22,23], bacterial growth inhibition [94] or in impairment of embryo-larval development of zebrafish [95]. However, a recent study indicated that rGO toxicity depended on the reduction pathway used to produce the material [96]. Studies previously cited highlighting a higher toxicity of rGO were performed using materials produced from acidification or using reducing agents such as hydrazine or ascorbic acid. In our case, it appears that thermal reduction in a H_2_ atmosphere of GO produced safer material with lower genotoxic potential. Initially, GO is composed of several oxygen-containing functional groups such as epoxy, hydroxyl and carboxyl groups, regardless of the production process [97]. XPS analysis performed on different materials produced allowed changes in the chemical surface composition of GO during reduction process. Therefore, functional groups such as epoxides were removed after annealing GO at 200 °C. These functions could clearly be responsible for the GO-induced genotoxic effects. Indeed, these epoxide functions are also produced in the liver by Benzo[a]pyrene metabolization and are well-known for being responsible for DNA adducts and damage induction [98]. Thus, contradictory results from the literature concerning the hazard potential of GBMs may possibly be explained by differences in the surface functions of the tested materials from one study to another. In our study, the fully-reduced GO produced after thermal reduction at 1000 °C led to a material with very few residuals of oxygenated surface groups and with a C/O ratio value comparable to the few layer graphene that was shown to be non-genotoxic towards *X. laevis* under similar experimental conditions [38], which is consistent with our observations and hypotheses.

## 4. Conclusions

According to results obtained in this work, we showed the importance of the nature of oxygen-containing functions of GBMs, especially the epoxide groups, in their hazard potential toward aquatic species. Indeed, GO is able to induce oxidative stress and inflammatory response at low concentrations, leading to mutagenic effects in vivo in *Xenopus laevis* tadpoles. At higher concentrations, the toxicity is reflected by disturbances in erythrocytic mitosis, resulting in accumulation of cells in G0/G1 phase. Thermal reduction of GO into rGO under our study conditions produced material that no longer induced oxidative stress, inflammation and genotoxic effects at low concentration. According to data from the literature, it appears that the reduction process used to produce rGO may determine the hazard potential of the reduced material. Thereby, although the thermal treatment of GO performed at 200 °C decreased the toxic potential of GO, reduction of material oxygen content through the methodology used in our study conditions appears to constitute a good strategy to produce a safer material for aquatic species [99].

## Figures and Tables

**Table 1:** Physico-chemical characteristics of graphene oxide (GO); reduced graphene oxide (rGO)200 and rGO1000. TEM: transmission electron microscope; HRTEM: high resolution TEM; BET: Brunauer-Emett-Teller; at. %: atomic %; GANF^®^: Grupo Antolin carbon nanofibers.

	GO	rGO200	rGO1000
Synthesis/production	GANF^®^ processed by Hummers’ method	Thermal treatment in hydrogen (5 L·h^−1^) at 200 °C (2 h)	Thermal treatment in hydrogen (5 L·h^−1^) at 1000 °C (2 h)
Catalyst	Ni, Fe, Co, Mn	None	None
Carbon content	69 ± 0.4 at. %	83.8 ± 0.5 at. %	98.5 ± 0.5 at. %
Oxygen content	31 ± 0.4 at. %	16.2 ± 0.3 at. %	1.5 ± 0.3 at. %
Number of layers (HRTEM)	1–5 [42,43]	1–5 [42,43]	1–5 [42,43]
Lateral size (TEM)	0.2 to 8 µm	0.2 to 8 µm	0.2 to 8 µm
Specific surface area (BET)	228 ± 6.8 m^2^·g^−1^	16 ± 0.5 m^2^·g^−1^	175 ± 5.2 m^2^·g^−1^

**Table 2:** Assignments of C1s XPS peaks for GO, rGO200 and rGO1000. Csp^2^: sp^2^ carbon; Sat.: shake-up satellites (π to π* transitions).

GO		rGO200		rGO1000	
Peak Assignment	at. %	Peak Assignment	at. %	Peak Assignment	at. %
Csp2 graphene	35.5	Csp2 graphene	64.5	Csp2 graphene	89.7
C–OH/C–O–C	24.7	C–OH/C–O–C	7.8	C–OH/C–O–C	0.6
C=O	2.5	C=O	5.8	C=O	0.5
O=C–O	5.3	O=C–O	1.3	O=C–O	0.1
Sat.	1.4	Sat.	4.5	Sat.	7.7

**Table 3:** Concentration of metals (Ni: nickel; Co: cobalt; Fe: iron; Mn: manganese) and polycyclic aromatic hydrocarbons (PAHs) released in the exposure medium at 10 mg·L^−1^ of GO. Among the 32 analyzed PAHs, only those with a concentration over the detection limit are listed.

	Metals Concentrations in the Medium (mg·L^−1^)		PAHs Concentrations in the Medium (µg·L^−1^)
**Ni**	35.5	**Naphtalene**	3.5 × 10^−4^
**Co**	24.7	**Acenaphtene**	2.5 × 10^−4^
**Fe**	2.5	**Phenanthrene**	3.2 × 10^−4^
**Mn**	5.3	**Fluoranthene**	2.4 × 10^−4^
		**Benzo(a)anthracene**	2.4 × 10^−4^
		**Chrysene**	2.5 × 10^−4^
		**Benzo(b+j)fluoranthene**	2.5 × 10^−4^
		**2-Methyl Naphtalene**	5.8 × 10^−4^

**Table 4:** Differential gene expression in *Xenopus larvae* liver (n = 5) after 12 days of exposure to GO, rGO200 and rGO1000 at 0.1 mg·L^−1^. For each condition, results are given as induction (>1) or repression (<1) factors compared to the negative control. Only statistically significant values are given; “-” indicates factors similar to control levels.

Functions	Genes	Genes Relative Expression		
Oxidative Stress Response		GO 0.1 mg·L^−1^	rGO200 0.1 mg·L^−1^	rGO1000 0.1 mg·L^−1^
	*gpx1*	5.84 ± 0.54	-	-
	*cat*	-	-	-
	*sod(Cu/Zn)*	2.76 ± 0.21	-	-
	*sod(Mn)*	2.48 ± 0.15	-	-
**Inflammation processes**	*pparγ*	5.71 ± 0.37	-	-
	*cox1*	3.65 ± 0.2	-	-
	*cox2*	-	-	-
	*lta4*	-	-	-
	*5-lox*	2.60 ± 0.17	-	-
**DNA repair**	*rad51*	-	-	-
	*mut*	-	-	-
	*odc*	-	-	-
**Detoxification**	*cyp1a1*	4.99 ± 0.53	-	-
	*tap*	19.09 ± 0.95	-	-
	*gst*	-	-	-

## Supplementary Materials

The following are available online at https://www.mdpi.com/2079-4991/9/4/584/s1, Figure S1: Gating strategy used in flow cytometry for analysis of erythrocyte cell cycle of *Xenopus laevis* tadpoles. Cells were gated to exclude debris (A). Single cells were gated using width and area parameters of IP fluorescence (B). Analysis of cells in G0/G1, S or G2/M phase was performed (C), Table S1: Accession numbers, functions and primer pairs (aUpstream primer; bForward primer) for the 25 *X. laevis* genes studied.
