# Supplementary material for: Thermal Reduction of Graphene Oxide Mitigates Its In Vivo Genotoxicity Toward Xenopus laevis Tadpoles
Source: Nanomaterials (Basel). 2019 Apr 9;9(4):584. doi: 10.3390/nano9040584 (PMC6523888; doi:10.3390/nano9040584)
Supplement: Supplementary file 1 [file nanomaterials-09-00584-s001.pdf]

Supplementary Materials

Thermal Reduction of Graphene Oxide Mitigates Its In Vivo Genotoxicity Toward *Xenopus laevis* Tadpoles

Lauris Evariste [1,*], Laura Lagier [1], Patrice Gonzalez [2], Antoine Mottier [1], Florence Mouchet [1], Stéphanie Cadarsi [1], Pierre Lonchambon [3], Guillemine Daffe [4], George Chimowa [3], Cyril Sarrieu [3], Elise Ompraret [3], Anne-Marie Galibert [3], Camélia Matei Ghimbeu [5], Eric Pinelli [1], Emmanuel Flahaut [2] and Laury Gauthier [1]

1 EcoLab, Université de Toulouse, CNRS, INPT, UPS, Toulouse, France
2 Univ. Bordeaux, UMR EPOC CNRS 5805, Aquatic ecotoxicology team, 33120 Arcachon, France
3 CIRIMAT, Université de Toulouse, CNRS, INPT, UPS, UMR CNRS-UPS-INP N°5085, Université Toulouse 3 Paul Sabatier, Bât. CIRIMAT, 118 route de Narbonne, 31062 Toulouse cedex 9, France
4 CNRS, Universite de Bordeaux, Observatoire Aquitain des Sciences de l'Univers, UMS 2567 POREA, Allee Geoffroy Saint Hilaire, F-33615, Pessac, France
5 Institut de Science des Matériaux de Mulhouse (IS2M), UMR 7360 CNRS - UHA, 15 rue Jean Starcky, BP 2488, 68057 Mulhouse cedex, France
* Correspondence: lauris.evariste@ensat.fr; Tel.: +33534323936

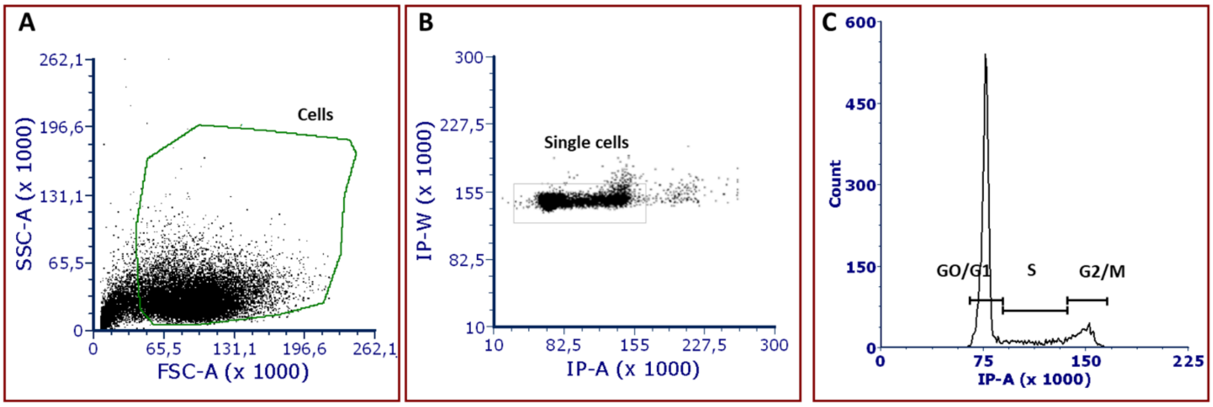

Figure 1. Gating strategy used in flow cytometry for analysis of erythrocyte cell cycle of Xenopus laevis tadpoles. Cells were gated to exclude debris (**A**). Single cells were gated using width and area parameters of IP fluorescence (**B**). Analysis of cells in G0/G1, S or G2/M phase was performed (**C**).

Table S1. Accession numbers, functions and primer pairs (aUpstream primer; bForward primer) for the 25 X. laevis genes studied.

Functions	Genes	Accession number	Primer (5′-3′)
**Oxidative stress response**			
	gpx1	NM_001095427	CTACCTGACCCTCCCCAAAC[a]<br>TTACCAACACCTGCCCTCA[b]
	cat	EF070606	GAGCAAGCGGAGAGATTCAA[a]<br>AGCACCAGTTTACCCACAGG[b]
	sod(Cu/Zn)	X51518	GTGTGCTGGCGGGAAG[a]<br>CCGGGGCTCCGTGATT[b]
	sod(Mn)	AY362041	TGTCAGTTGGAGTCCAGGG[a]<br>AGTTGATAACATTCCAGATCGCTTT[b]
**Inflammation processes**	*pparγ*	M84163	AAAGACGGGGTGCTCGTAG[a]<br>TGAAGTCGGAGAAGGGTTTG[b]
	cox1	AB278691	GGATGGGACGGGGTTTCATT<br>TCCTGCCCTACTGCGAATTG
	cox2	NM_001093477	GGCCATGGGGTTGATCTCAA[a]<br>CTGGAACATGGGGTGGGTAC[b]
	lta4	NM_001091767	TTGTTACCCCAACTGTGCTG[a]<br>GTTTTCCCAGGTTTCATTCG[b]
	5-lox	BG656985	AGTCAGCACGCAGTTGTGAA[a]<br>ACACAACTCCAAGGTGCCAG[b]
**DNA repair**	*rad51*	BC108486	TCAAGAAACTGGAGGACGCC[a]<br>CGCCTCTGGTGAAACTCAGT[b]
	mut	BE681219	TATGAGGACTGAGAAAACAGAAGCA[a]<br>AGTTACAAACACTTGGAGCTGAT[b]
	odc	NM_001086698	TCAGGAGAGCGGAATGGA[a]<br>GGTCCCAAGGCTAAAGTTGC[b]
**Detoxification**	*cyp1a1*	NM_001097072	TGCTGAATGTTTCCTGGGAGT[a]<br>CCCCTTGCACTTTTGGTTCC[b]
	tap	AY204551	TTGCTGTCGGTGGGGC[a]<br>CAGTGTTGATTCGGTGGG[b]
	gst	AJ489617	CGTCCTCACCTATTTCCCCG[a]<br>CCTGAAACTGAGGCAGCTGA[b]
**References**	gapdh	NM_001087098	ACCCAGAAGACAGTGGATGG[a]<br>CAGTTGAGGCGGGAATAATG[b]
	B actin	BE490926	ATTGAGCCACCAATCCAGG[a]<br>ACAAGTGTTGGAATGTGCG[b]
